# Supplementary material for: A repressive regulatory cascade shapes temporal patterning of activity-regulated gene expression in a defined sensory neuron type
Source: bioRxiv. 2026 Jul 29:2026.05.14.725236. Preprint. [Version 3] doi: 10.64898/2026.05.14.725236 (PMC13192744; doi:10.64898/2026.05.14.725236)
Supplement: Supplementary file 1 [file media-1.pdf]

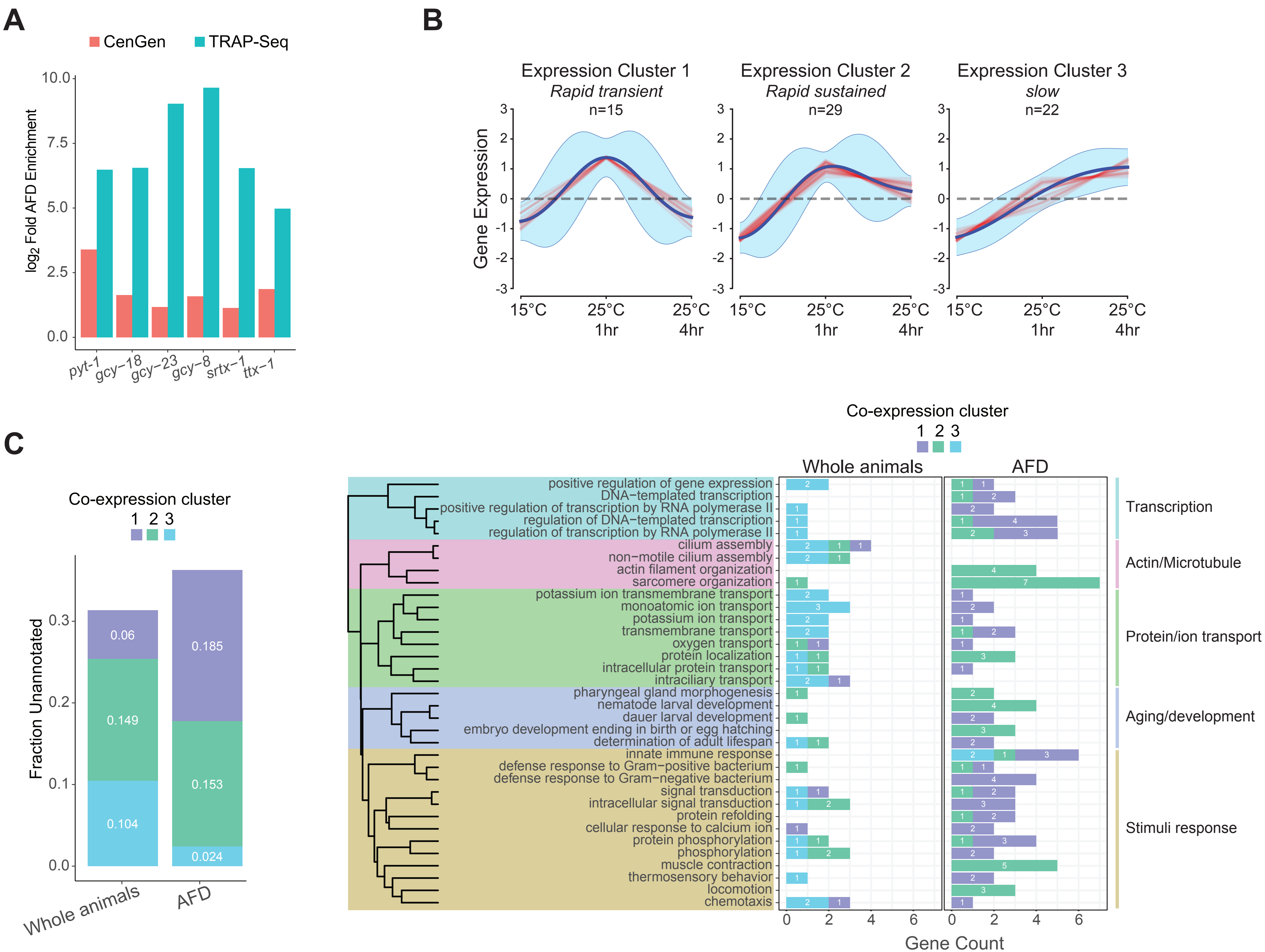

Bates et al., Figure S1

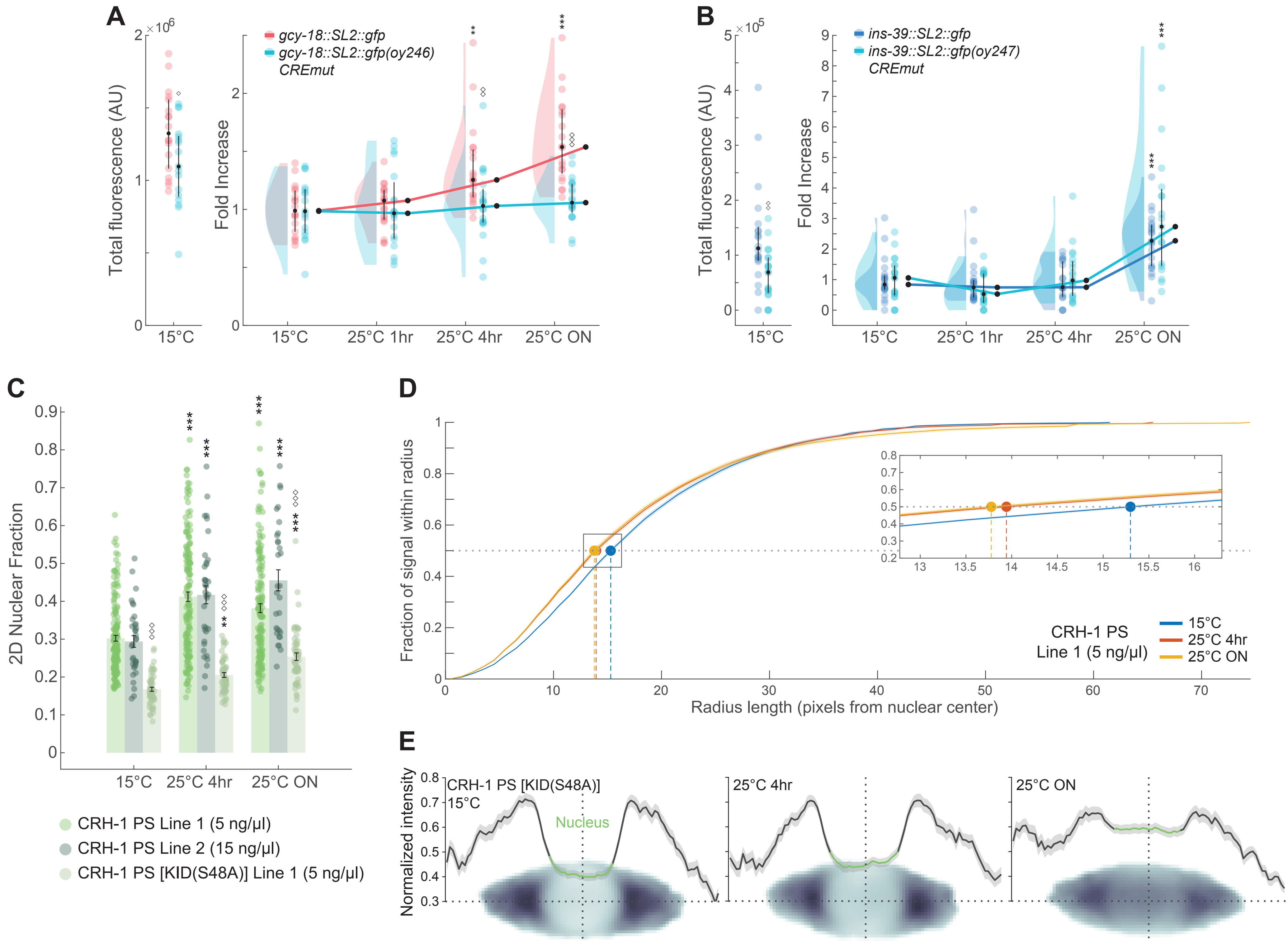

Bates et al., Figure S2

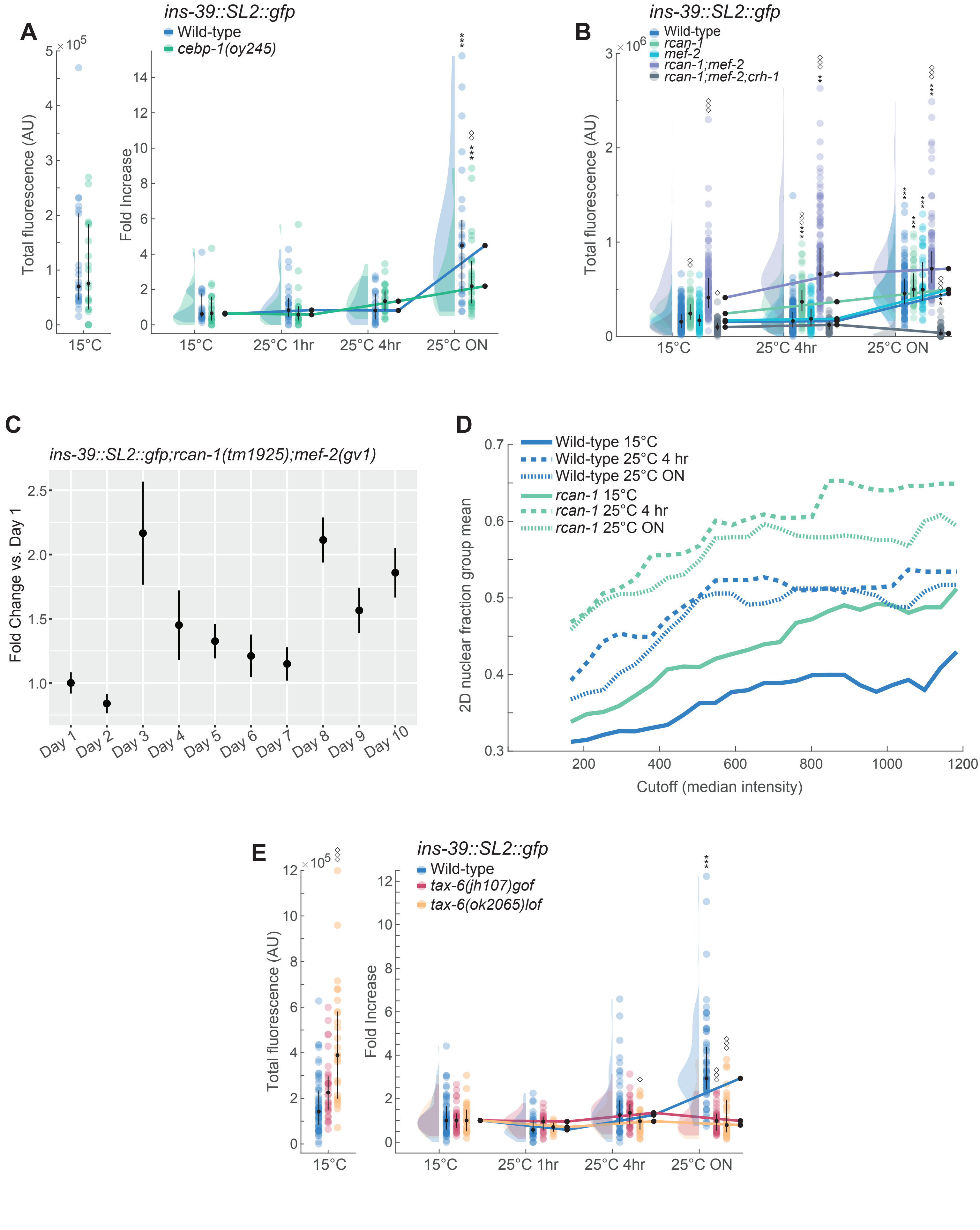

Bates et al., Figure S3

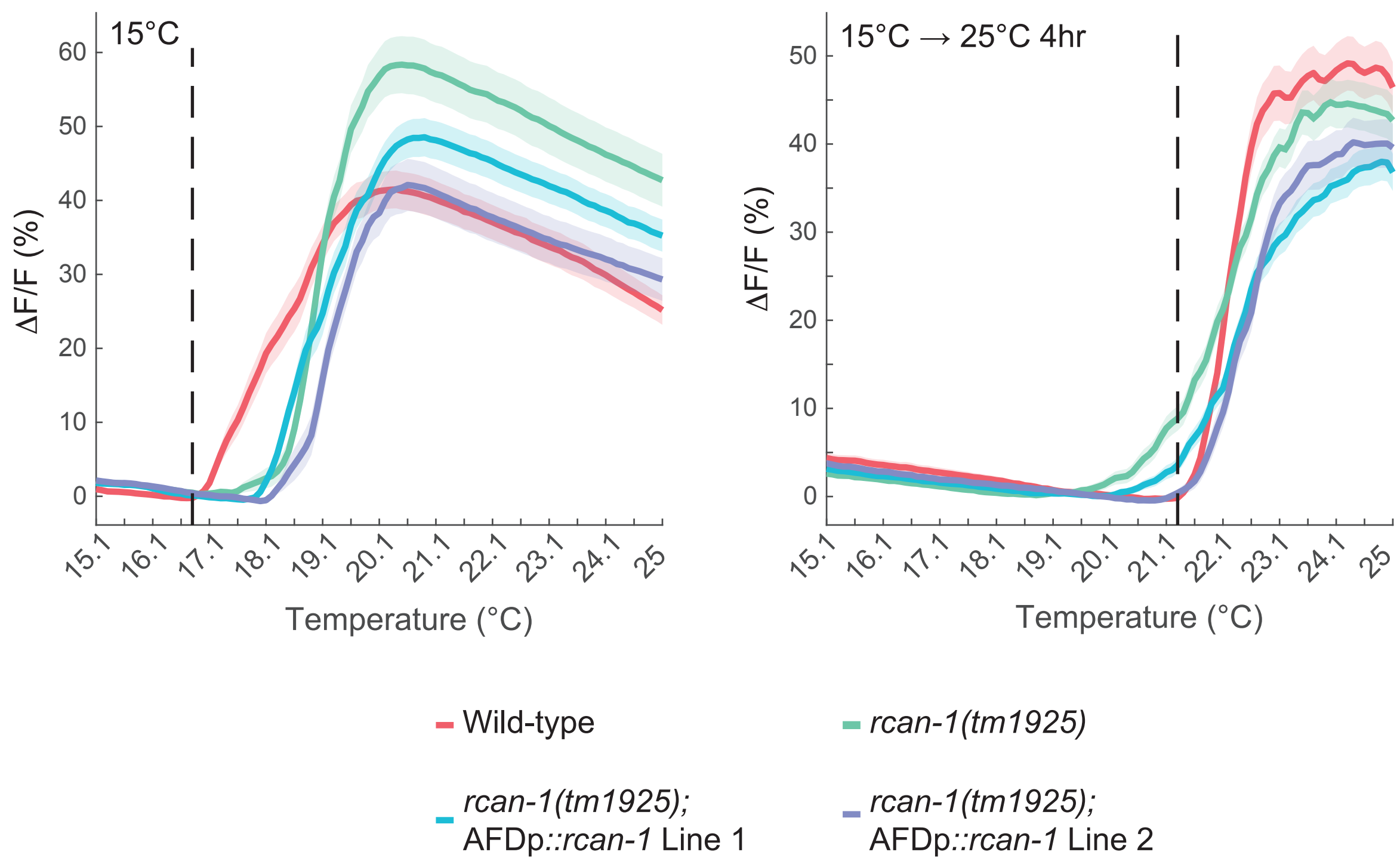

Bates et al., Figure S4
